# Incidence and perception of nursing students’ academic incivility in Oman

**DOI:** 10.1186/s12912-017-0213-7

**Published:** 2017-04-21

**Authors:** Jansi Natarajan, Joshua Kanaabi Muliira, Jacoba van der Colff

**Affiliations:** 1 Department of Fundamentals & Administration College of Nursing, Sultan Qaboos University, P. O. Box 66 Al Khod, Muscat, Oman; 2 Department of Adult Health & Critical Care College of Nursing, Sultan Qaboos University, P. O. Box 66 Al Khod, Muscat, Oman; 3 Ministry of Health, Directorate of Nursing and Midwifery Government of Sultanate of Oman, Muscat, Oman

**Keywords:** Nursing education, Safety, Professionalism, Incivility, Undergraduate

## Abstract

**Background:**

The incidence of incivility in nursing education is increasing in most countries and it is affecting the culture of safety and the teaching-learning processes. Despite reports of increasing trends, little is known about nursing students’ academic incivility in the Middle East. This study aimed at exploring the perceptions and extent of academic incivility among nursing students (NS) and nursing faculty members (NF) in a university based undergraduate nursing program in Oman.

**Methods:**

A quantitative cross sectional survey was used to explore NS academic incivility from the perspective of NS and NF in a public university in Oman. Data was obtained from a sample of 155 NS and 40 NF using the Incivility in Nursing Education Survey.

**Results:**

There was agreement between NS and NF on the majority of behaviors perceived to be disruptive. The incidence of NS academic incivility was moderate. The most common uncivil behaviors were acting bored or apathetic in class, holding conversations that distract others in class, using cell phones during class, arriving late for class, and being unprepared for class. There were significant differences between NF and NS perceived incidence of uncivil behaviors such as sleeping in class (*p* = 0.016); not paying attention in class (*p* = 0.004); refusing to answer direct questions (*p* = 0.013); leaving class early (*p* = 0.000); cutting or not coming to class (*p* = 0.024); and creating tension by dominating class discussions (*p* = 0.002).

**Conclusion:**

Student academic incivility is moderately present in nursing education in Oman, and this may have implications in terms of the future of the profession and patient care. There is need for more streamlined policies and strategies to curtail the incidence of academic incivility and to maintain safe and effective learning environments.

## Background

Incivility is increasing among nursing students (NS) and it is one of the problems affecting nursing education in different countries [1, 2]. Professional organizations such as the National League for Nursing are constantly highlighting the importance of incivility in nursing [3]. The defining elements of civility include respect for others, honoring differences, listening, seeking common ground and engaging in social discourse and appreciating its relevance [4]. Incivility on the other hand includes disrespect for others, the inability or unwillingness to listen to other’s points of view or to seek common ground, and not appreciating the relevance of social discourse. In nursing education incivility has been defined as rude or disruptive behaviors which often results in psychological or physiological distress for the people involved, and if left unchecked, may result into ominous situations [5].

The other terms that have been used to refer to student incivility in nursing education include difficult students, difficult student situations, inappropriate student behaviors, lateral violence, and disruptive behaviors [6]. Some of the behaviors which nursing faculty members in other countries have reported to show incivility include making disapproving groans, making sarcastic remarks or gestures, and cheating on examinations [7]. The main factors used to explain the increasing uncivil conduct among NS are stress, attitudes of entitlement, and nursing faculty (NF) attitude of superiority [8]. Studies conducted among NS and NF in countries such as USA and China show that NS incivility is common [2, 8]. In USA the common NS uncivil behaviors include arriving late for class, holding distracting conversations, being unprepared for class, leaving class early and not turning up for class [8].

In China the common uncivil student behaviors reported by NF include cheating on exams and quizzes; dominating class; using cell phones during class; holding distracting conversations; demanding make up exams; and arriving late for class [2]. The source of incivility is from both the NS and NF [9], and it can take place in the classrooms [10] and the clinical settings [11]. The NS know that incivility exists and they feel that nursing faculty members contribute to its escalation [12].

Understanding the prevalence, source and forms of incivility in nursing education is critical because of its implications for learning outcomes and the well-being of NF [13]. Incivility in nursing education undermines the culture of safety, and the intimidation created by such behaviors leads to an environment of hostility and disrespect, all of which reduces morale, diminishes patient safety, and increases staff turnover, distraction, and number of errors [14]. Studies conducted in USA show that overtime NS incivility negatively impacts NF leading to problems such as decreased self-esteem, loss of confidence in teaching ability, loss of sleep, loss of time, withdrawal from teaching jobs [1] and high stress levels [9]. It is likely that as the population of NS becomes more diverse, the problem of incivility will continue to be a challenge and will be more difficult to understand because of the cultural differences and lack of knowledge about warning signs [15].

In Oman most of the NF are foreigners (expatriates from other countries) and they have different cultures from that of their NS. The NS receive free education from primary school until the university. This availability of free education and expatriate teachers is likely to increase a sense of entitlement and cultural misunderstandings, both of which can elicit incivility. Our experience as NF in Oman, and informal feedback from colleagues and clinical preceptors shows that some NS are difficult to teach because of their uncivil behaviors. Similarly a study conducted in Kuwait found academic incivility to be at moderate prevalence levels among college students [16].

There are no other studies from Middle Eastern countries that have reported about academic incivility. Therefore there is need for more studies in the Middle East region to document the extent, source, and nature of NS incivility and the associated factors. The aim of this study was to explore the perceptions and extent of academic incivility among NS and NF in a University based Bachelor of Science in Nursing (BSN) program in Oman.

## Methods

A cross sectional descriptive design was used to collect data from NS and NF in a university based BSN program. At the time of the study, the BSN program was the only program at this level in a government institution and the largest in Oman. The estimated NS population was 400 and the college had 58 NF. The NS admitted in the BSN program come from different regions of Oman and they all get free education paid for by the government. The annual college student intake is comprised of 100 direct entry students (from high school) and 20 bridging students (qualified with a diploma in nursing) in the 4-year and 2-year nursing program, respectively.

### Sample

The participants for this study were the NS and NF in the BSN program. Examining the phenomenon of student academic incivility from the NS and NF perspectives provided a balanced view of the sources and extent of incivility. All potential participants received emails publicizing the study. A total of 200 NS and 50 NF were directly approached by the research assistant and requested to participate in the study. The potential participants were approached in their offices (NF) and during class break (NS). The number of NS and NF who agreed to participate in the study were 155 and 40, respectively. These rates of participation represent a response rate of 78 and 80% for NS and NF, respectively. The convenient sampling strategy was used and was the most feasible in order to obtain an adequate sample in view of the rigorous and tight scheduling of classes, clinical placements, and NF teaching and other responsibilities.

The NS had to meet the inclusion criteria of being a: BSN student who is at least 18 years of age; spent at least one year in the BSN program; officially registered and studying at university; completed at least one clinical nursing course; and has not yet graduated from the university. The inclusion criteria for NF were: worked at the college for at least 12 months; and having responsibilities which require getting into direct contact with NS. The NF were excluded if they had not completed at least 12 months of employment at the college or if their work involved only administrative responsibilities which do not require coming into direct contact with NS.

### Study instrument

A self-administered questionnaire (SAQ) written in English was used to collect data. The language of instruction in the nursing program is English and all materials used for teaching are written in English. The main variables that were measured were demographic characteristics and academic incivility. The SAQ for NS was comprised of three sections (demographic characteristics, experiences related to incivility and incivility in nursing education survey). The SAQ for NF was comprised of three sections (demographic characteristics; experiences related to incivility and incivility in nursing education survey).

In this study incivility was defined as rude or disruptive behaviors in the academic environment that may result in psychological or physiological distress for NF and NS involved and if left unaddressed may progress into a threatening situation. Academic incivility was measured using the 2010 version of the Incivility in Nursing Education (INE) survey. The INE is a valid and reliable tool which was designed to measure NS and NF perceptions of incivility and frequency of uncivil behaviors [8]. The INE survey is divided into two sections [17].

The first section of the INE contains 122 items focusing on disruptive and threatening behaviors. The participants are first required to indicate whether a behavior is disruptive with a response of “yes” and “no”. And then on the same item the participants are required to rate how often they have experienced or seen the behavior in the academic environment in the past 12 months using a 4-point Likert Scale (Always = 4; Usually = 3; Sometimes = 2; and Never =1). The 4-point Likert scale was used to determine the incidence of specific acts of incivility. The mean score for each behavior was calculated for the whole sample to determine the incidence of its occurrence. In other studies the INE Cronbach’s alpha for inter-item coefficients were found to range from 0.80 to 0.84 [5, 8]. In the current study the INE Cronbach’s alpha was 0.74. The second section of the INE survey has Open ended questions which give participants to provide more information about the incivility observed, experienced or perceived. The results from the open ended questions (qualitative data) are not included in this report.

### Data collection procedure

The study was advertised to all NS and NF through emails and fliers. The investigators were given permission to access the list of all NF and NS who had completed at least one clinical course. The study recruited two research assistant (RA) and one was assigned to collect data from NF. The RA approached NF when they are in their offices. The offices of NF were located using the college directory available on the website. If the NF was not in the office, the RA made three attempts at different times of the week using the NF schedule posted on the door as a guide to determine the most appropriate time to find the participant. In cases where the NF was busy an appointment was taken to come back later to conduct the data collection. The offices whose occupants agreed or refused to participate were crossed out on the directory list to avoid repeat data collection.

A second RA was assigned to collect data from NS during class breaks. The class schedule available on the college website was used to determine when each class starts and ends. The NS that agreed or refused to participate in the study were crossed out on the list obtained to avoid repeat data collection. On completion of data collection, the marked list was stored in a different location from the completed questionnaires to maintain confidentiality and privacy of NS. The data collection for all participants took place in a designated private room or the NF office.

### Data analysis

Data analyses were conducted using SPSS for Windows (SPSS-22.0). The data was assessed for normality using the Shapiro-Wilk statistics and the results (W = 0.934; *p* = 0.325) demonstrate normal distribution. Descriptive statistics (percentage, mean, standard deviation, and *p*-values) were used to summarize NS and NF perceptions of academic incivility and to determine the most common uncivil behaviors. The independent samples *t*-test for uncorrelated means was used to determine differences between NS and NF perceptions. The level of statistical significance for all statistical analyses was set at 0.05 (two-tailed).

## Results

### Characteristics of the nursing faculty

The results summarized in Table 1 show that the majority of NF were female (72%) and were qualified with a master or PhD (85%). The different academic ranks of the NF were represented in the sample starting from teaching assistants (8.1%), lecturers (70.3%), assistant professors (16.2%) and associate professors (5.4%). The nursing faculty members are from diverse countries including Oman (28.6%), India (28.6%), the Philippines (20%), Jordan (11.4%) and Uganda (8%).

**Table 1:** Characteristics of nursing faculty

Characteristic	Category	Frequency (*n* = 40)	Percent
Age in years (Mean = 36.16, SD = 9.77)	18–21	3	7
	22–30	10	23.2
	>30	30	69.8
Gender	Male	9	28.1
	Female	23	71.9
Academic Qualification	Bachelors	6	15
	Masters	24	60
	Doctorate	10	25
Number of years as a nurse educator (Mean = 11.56; SD = 8.34)	<5	8	22.2
	6–15	21	58.3
	16–25	4	11.2
	26–30	3	8.3
Number of years in current position (Mean = 4.63; SD = 3.32)	<5	15	53.6
	6–10	7	25
	11–15	2	7.1
	16–20	4	14.3
Average No of students in a theory class (Mean =35.31; SD = 20.629)	<20	7	17.1
	21–40	10	24.4
	41–60	8	19.5
	>60	16	39
Average number of students at a time in Clinical (Mean = 7.55: SD = 8.38)	<20	32	76.2
	21–40	3	7.1
	41–60	2	4.8
	>60	5	11.9
Level of nursing courses in the BSN Program you commonly teach	Third level	10	37
	Fourth level	12	44.4
	All levels	5	18.5

### Characteristics of the nursing students

The results presented in Table 2 show that the majority of NS were between the age of 18 to 21 years (71.1%) and with a cumulative grade point above 2 out of 4 (97.5%) and were in the third year of the nursing program. A large number of students were familiar with the discipline expected of healthcare professional since they had at least one family member who was a nurse or other healthcare professions.

**Table 2:** Characteristics of Nursing Students

Characteristic	Category	Frequency (*n* = 155)	Percent
Age in years (Mean = 22.19; SD = 3.59)	18–21	118	71.1
	22–30	32	19.3
	>31	16	9.6
Gender	Male	43	33.3
	Female	86	66.7
Cumulative grade point average (self-reported)	0–2	4	2.6
	2.1–4	151	97.4
Year of study in the BSN program	Two	23	18.4
	Three	69	55.2
	Four	33	26.4
Number of close friends in the college	<20	99	67.8
	>20	47	32.2
Has a family member who is a nurse	Yes	77	50.3
	No	76	49.7
Has a family member who is a doctor or any other health profession	Yes	47	30.3
	No	108	69.7

### Faculty and students’ opinion about disruptive behaviors

The participants were asked to state whether each of the student behavior presented in Table 3 is disruptive or not. The majority (>50%) of NS and NF agreed that most of the stated behaviors were disruptive. There were no significant differences between the NS and NF regarding the behavior perceived to be disruptive except on two aspects. Significantly more NF compared to NS felt that acting bored or apathetic in class (Ӽ^2^ **=** 5.99) and holding conversations that distract other students in class (Ӽ^2^ **=** 5.79) are disruptive behaviors.

**Table 3:** Students’ and faculty members’ opinion about disruptive student behaviors

Student Behaviors	Response	Faculty (*n* = 40) f (%)	Student (*n* = 155) f (%)	Chi Square (Ӽ^2^) and *p*-value
Acting bored or apathetic	Yes	34(81)	89(60.5)	Ӽ^2^ = 5.99 *p* = 0.014
	No	8(19)	58(39.5)	
Making disapproving groans	Yes	25(64.1)	80(56.7)	Ӽ^2^ = 0.68 *p* = 0.41
	No	14(35.9)	61(43.3)	
Sleeping in class	Yes	28(71.8)	96(64)	Ӽ^2^ = 0.83 *p* = 0.36
	No	11(28.2)	54(36)	
Not paying attention in class (doing work for other courses, reading a newspaper, not taking notes)	Yes	32(80)	38(25.5)	Ӽ^2^ = 0.52 *p* = 0.47
	No	8(20)	11(74.5)	
Holding conversations that distract you or other students	Yes	37(90.2)	106(72.1)	Ӽ^2^ = 5.79 *p* = 0.02
	No	4(9.8)	41(27.9)	
Refusing to answer direct questions	Yes	17(43.6)	68(45.9)	Ӽ^2^ = 0.07 *p* = 0.79
	No	22(56.4)	80(54.1)	
Using computer during class for purposes not related to the class	Yes	20(51.3)	81(54.4)	Ӽ^2^ = 0.12 *p* = 0.73
	No	19(48.7)	68(45.6)	
Using cell phones or pagers during class	Yes	36(87.8)	112(74.2)	Ӽ^2^ = 3.39 *p* = 0.07
	No	5(12.2)	39(25.8)	
Arriving late for class	Yes	32(80)	106(71.2)	Ӽ^2^ = 1.52 *p* = 0.218
	No	8(20)	45(29.8)	
Making sarcastic remarks or gestures (staged yawning, eye rolling)	Yes	30(73.2)	84(57.1)	Ӽ^2^ = 3.45 *p* = 0.063
	No	11(26.8)	63(42.9)	
Leaving class early	Yes	28(68.3)	81(54)	Ӽ^2^ = 2.69 *p* = 0.10
	No	13(31.7)	69(46)	
Cutting (not coming for) class	Yes	25(62.5)	92(60.9)	Ӽ^2^ = 0.03 *p* = 0.86
	No	15(37.5)	59(39.1)	
Being unprepared for class	Yes	31(79.5)	97(64.7)	Ӽ^2^ = 3.11 *p* = 0.078
	No	8(20.5)	53(35.3)	
Creating tension by dominating class discussions	Yes	25(64.1)	84(56)	Ӽ^2^ = 0.83 p = 0.36
	No	14(35.9)	66(44)	
Cheating on exams or quizzes	Yes	23(57.5)	94(62.3)	Ӽ^2^ = 0.30 *p* = 0.58
	No	17(42.5)	57(37.7)	
Demanding make up exam, extensions, grade changes, or other special favors	Yes	31(75.6)	88(59.9)	Ӽ^2^ = 3.42 *p* = 0.06
	No	10(24.4)	59(40.1)	

### Experiences with uncivil student behaviors

The NS and NF were asked to identify the threatening NS behaviors they have experienced or which have happened to someone they know in past three months. The results presented in Table 4 shows the most common uncivil NS behaviors experienced by NF were general taunts or disrespect to other students (15.4%), property damage (18.4%), harassing comments directed at faculty (30.8%), general taunts or disrespect to NF (38.5%) and challenges to faculty knowledge or credibility (42.5%). The most common uncivil NS behaviors experienced by NS were harassing comments directed at students (21.3%), general taunts or disrespect to NF (29.9%), general taunts or disrespect to other NS (30.1%), threats of physical harm against other NS (34.2%) and challenges to faculty knowledge or credibility (36.7%). There were no significant differences between NS and NF experiences of uncivil student behaviors except on one behavior of general taunts or disrespect to faculty (Ӽ^2^ **=** 1.06).

**Table 4:** Students’ and faculty members’ experience with uncivil students behaviors

Student Experiences of Incivility	Response	Faculty (*n* = 40) f (%)	Student (*n* = 155) f (%)	Chi Square (Ӽ^2^), *p*-value
General taunts or disrespect to other students	Yes	6(15.4)	47(30.1)	Ӽ^2^ = 3.43 *p* = 0.06
	No	33(84.6)	109(69.9)	
General taunts or disrespect to faculty	Yes	15(38.5)	46(29.9)	Ӽ^2^ = 1.06 *p* = 0.03
	No	24(61.5)	108(70.1)	
Challenges to faculty knowledge or credibility	Yes	17(42.5)	55(36.7)	Ӽ^**2**^ = 0.46 *p* = 0.50
	No	23(57.5)	95(63.3)	
Harassing comments (racial, ethnic, gender) directed at students	Yes	6(15.8)	33(21.3)	Ӽ^2^ = 0.55 *p* = 0.46
	No	32(84.2)	123(78.8)	
Harassing comments (ethnic, gender) directed at faculty	Yes	12(30.8)	29(19.3)	Ӽ^2^ = 2.383 *p* = 0.12
	No	27(69.2)	121(80.7)	
Vulgarity directed at students	Yes	4(1.05)	30(19.4)	Ӽ^2^ = 2.69 *p* = 0.26
	No	34(89.5)	108(78.3)	
Vulgarity directed at faculty	Yes	5(13.2)	23(16.8)	Ӽ^2^ = 0.29 *p* = 0.59
	No	33(86.8)	114(83.2)	
Inappropriate emails to other students	Yes	5(13.5)	20(12.8)	Ӽ^2^ = 0.01 *p* = 0.91
	No	32(86.5)	136(87.2)	
Inappropriate emails to faculty	Yes	5(13.2)	16(10.7)	Ӽ^2^ = 0.19 *p* = 0.66
	No	33(86.8)	134(89.3)	
Threats of physical harm against other students	Yes	4(10.5)	13(34.2)	Ӽ^2^ = 0.16 *p* = 0.69
	No	34(89.5)	140(91.5)	
Threats of physical harm against faculty	Yes	4(10.5)	8(5.2)	Ӽ^2^ = 1.45 *p* = 0.23
	No	34(89.5)	145(94.8)	
Property damage	Yes	7(18.4)	17(11.1)	Ӽ^2^ = 1.48 *p* = 0.22
	No	31(81.5)	136(88.9)	
Statements about having access to weapons	Yes	3(7.9)	8(5.3)	Ӽ^2^ = 0.36 *p* = 0.55
	No	35(92.1)	142(94.7)	

### Incidence of uncivil student behaviors

The participants were asked to rate how often they have experienced or seen specific uncivil NS behaviors in the past 12 month using a 4 point Likert scale. Uncivil behaviors with a mean score of above 2 and above were considered to be significant incivility. The average rating by both the NS and NF of the incidence of all the 16 behaviors considered was 2.08 (moderate incidence) showing that they were collectively occurring more than sometimes. The results presented in Table 5 show that, among both NF (*n* = 40) and NS (*n* = 155) the behaviors with the highest incidence include acting bored or apathetic (94.87%), holding conversations that distract others in class (92.31%), using cell phones or pagers during class (98.97%), arriving late for class (96.92%) and being unprepared for class (97.44%). There were significant differences between NF and NS rating of uncivil behaviors such as sleeping in the class; not paying attention in the class; refusing to answer direct questions; using computers during class for purposes not related to the class; leaving class early; cutting or not coming to class; and creating tension by dominating class discussions.

**Table 5:** Incidence of uncivil student based on experiences and observation of faculty members and students

Student Behaviors	Participant	N	M	SD	SEM	t	df	*p*
Acting bored or apathetic	Faculty	42	2.286	0.774	0.119	0.889	183	0.375
	Student	143	2.168	0.750	0.063			
Making disapproving groans	Faculty	39	1.821	0.756	0.121	−1.332	168	0.185
	Student	131	2.000	0.734	0.064			
Sleeping in class	Faculty	40	1.800	0.823	0.130	−2.429	187	0.016
	Student	149	2.168	0.857	0.070			
Not paying attention in class	Faculty	40	2.000	0.716	0.113	−2.965	70.01	0.004
	Student	148	2.392	0.830	0.068			
Holding conversations that distract you or other students	Faculty	42	2.119	0.705	0.109	−1.669	84.61	0.099
	Student	138	2.341	0.892	0.076			
Refusing to answer direct questions	Faculty	39	1.590	0.715	0.115	−2.501	182	0.013
	Student	145	1.966	0.861	0.072			
Using computer during class for purposes not related to the class	Faculty	37	1.460	0.605	0.100	−2.799	176	0.006
	Student	141	1.879	0.858	0.072			
Using cell phones or pagers during class	Faculty	42	2.381	0.936	0.144	−1.429	191	0.155
	Student	151	2.616	0.944	0.077			
Arriving late for class	Faculty	39	2.256	0.715	0.115	−1.510	70.361	0.136
	Student	150	2.460	0.872	0.071			
Making sarcastic remarks or gestures (staged yawning, eye rolling)	Faculty	42	1.810	0.707	0.109	−1.643	178	0.102
	Student	138	2.044	0.836	0.071			
Leaving class early	Faculty	43	1.605	0.541	0.082	−4.020	188	0.000
	Student	147	2.102	0.756	0.062			
Cutting (not coming for) class	Faculty	40	1.975	0.660	0.104	−2.301	68.42	0.024
	Student	146	2.253	0.741	0.061			
Being unprepared for class	Faculty	42	2.500	0.707	0.109	−1.014	188	0.312
	Student	148	2.649	0.872	0.072			
Creating tension by dominating class discussions	Faculty	40	1.700	0.758	0.120	−3.198	177	0.002
	Student	139	2.158	0.810	0.069			
Cheating on exams or quizzes	Faculty	40	1.775	0.800	0.127	−1.266	178	0.207
	Student	140	1.957	0.804	0.068			
Demanding make up exam, extensions, grade changes, or other special favors	Faculty	42	2.095	1.031	0.159	-.405	178	0.686
	Student	138	2.159	0.856	0.073			

*M* Mean, *SD* Standard Deviation, *SEM* Standard Error Mean, *t t*-test statistic, *df* degrees of freedom, *p* p-value

## Discussion

This study sought to explore the perceptions and extent of nursing students’ (NS) academic incivility in a public University in Oman because there was no literature or studies which have addressed this problem in the Middle East region. The findings show that the incidence of NS academic incivility was moderate and a general agreement between the NF and NS on behaviors that are considered to be disruptive. These results were remarkable considering the fact that many NS reported that they had a family member who was a nurse (50.3%) and/or a doctor or other healthcare professions (69.7%), and therefore familiar with the role expectations of health care professionals and need for civil behaviors.

The presence moderate NS academic incivility despite familiarity with the social roles of health care providers, and the self-report method of data collection used in this study, leads us to suspect that the incidence may be higher than reported. The fact that most of the NS compared to NF did not feel that acting bored or apathetic in class and holding conversations that distract themselves or other students in class are disruptive behaviors implies that they are also likely to find it difficult to stop such behaviors. The most common uncivil NS behaviors found by this study were acting bored or apathetic, holding conversations that distract others in class, using cell phones or pagers during class, arriving late for class and being unprepared for class. These findings are partly similar to those reported in a study of NS in USA [17]. The above common uncivil NS behaviors in way indicate the trends of the millennial generation which tends to be socialized through technology and peers and not adults and elders who can impart civility [18].

On the other hand, the NF are usually older and more likely to view NS behaviors such using cell phones in class, holding conversations in class, and distracting others in class as significant incivility and a direct assault on their professional capability [19]. The differences between NS and NF perceptions of what is considered uncivil can also lead to underestimation of the incidence of some behaviors. For instance there were significant differences between NF and NS incidence rating of behaviors such as sleeping in the class; not paying attention in the class; refusing to answer direct questions; using computers during class for purposes not related to the class; leaving class early; cutting or not coming to class; and creating tension by dominating class discussions. This tells us that NS perceive these behaviors to be civil in the academic environment.

The trends of academic incivility are going to continue to be a challenge unless nursing educators develop innovative ways of addressing it. Incivility is an indicator of the short falls of nursing education because one of the universal goals of higher education is to promote civility and respect, and to create scholars, working professionals, and good citizens [20]. The uncivil behaviors reported in this study are all serious and can have a major negative impact on the learning process and professional aspirations of others in the classroom. Indeed both the students and faculty members are reported to be annoyed by student activities which are not related to learning and which disrupt the learning process [21]. The uncivil NS behaviors reported in this study are similar to those documented in other earlier studies [22–24]. Our findings are also similar to those which have been reported about NS incivility in the online learning environment that show a somewhat different perceptions of the extent of incivility experienced but agreed on identification of some uncivil behaviors [25]. However it should be noted that until today very few interventional studies have addressed the problem of NS incivility.

The NF play a major role in contributing to NS incivility [3]. It has been suggested that sometimes disruptive behaviors stem from a lack of clear learning and/or professional behaviors [8]. This implies that practices such as engaging students in an open discussion to establish classroom and clinical norms during the first week of classes, may help to curtail incivility [8]. The use of interactive teaching methods to boost students’ learning abilities and relationships between NF and NS has also been suggested [26]. Other reports have recommended the use of a preventive approach, while simultaneously delineating progressive disciplinary measures to proactively curb incivility. This recommendation is consistent with the mindset that posits prevention as the cornerstone of effective incivility management [27, 28]. The preventive approach requires setting clear expectations and using strategies such as distributing syllabi whose contents is designed to address administrative areas such as course objectives, evaluation methods, exam and attendance policies, and repercussions associated with acts of incivility [29].

Incivility can also be addressed by developing new policies. One of the policy approaches that can be used include formulation of a bill of rights for both students and faculty, and dissemination and referring to it in the course syllabi to help students to become acquainted with the standards to which both NF and NS are held [27]. The other potential strategies for handling incivility include building respectful and trusting relationships between NF and NS, outlining and discussing ethical behaviors during student orientation programs, incorporating honor codes into all nursing classes, and avoiding the culture of blame [29]. Interventions such as civility journal clubs have also been found to lead to positive changes in attitudes and behaviors related to civility of NS, and the students participating in such activities are more likely to be helpful to their peers [30].

However, one of the most important parts in the process of curtailing incivility is the support given to the NF when dealing will student incivility. Available studies show that NF many times feel a lack of support within their departments for dealing with NS incivility and feel threatened when confronting students who demonstrate uncivil behavior [31]. The lack of support is compounded by the fact that in some nursing institutions, there is no gold standard for managing NS incivility. For such institutions some of the solutions that could be adopted include initiating academic incivility zero-tolerance policies, consistently enforcing codes of conduct [22]; increasing faculty support and faculty development activities focusing on incivility [32]. Providing NF with training for effective communication and conflict negotiation, and role-modeling professionalism have been suggested as essential strategies for combating academic incivility [33]. All the above suggestions are mostly based on professional experiences of NF, but not empirical research.

Research studies addressing potential solutions to NS incivility are conspicuously lacking. There is need for more research studies to determine the relationship between NF and NS incivility, and the effectiveness of specific strategies towards curtailing or preventing incivility. Longitudinal investigations are also needed to examine the relationship between NS incivility and behaviors seen at the workplace such as bullying or lateral violence and professional incompetence. A deeper understanding of incivility and its relationship to patient safety and professional quality of life can help us to improve patient outcomes and nurses’ job performance.

### Implications for nursing education

The extent, perception and impact of NS academic incivility is likely to continue to increase and to be different from one setting to another. The findings of this study lead us to conclude that there is need for formal policies and continuous monitoring focusing on NS incivility. In order to have a good working knowledge of the factors underlying incivility and to calibrate interventions to address incivility, nurse educators need to conduct regular studies and surveillance. The findings of such studies will continuously update us on the magnitude of the problem, effective interventions and will allow for trending and comparisons. A part from policy and monitoring, we recommend using interventions such as a deliberate faculty development activities on NS academic incivility. The use deliberate training programs for faculty members will help to alert NF about the sources, preventive measures, interventions, responsibilities and potential impact on safety culture, teaching and learning outcomes and the profession at large.

### Limitations

The findings of this study should be considered in view of the following limitations, which may affect the generalizability of its results. The study is based on data collected from a convenient sample of NF and NS drawn from one nursing institution and program in Oman. The cultural background of NS in Oman compared to that of NS in other regions of the world, may have an influence on their behaviors and perceptions. It is also important to acknowledge that the study used a self-report method of data collection and the INE survey used to measure NS incivility has not been previously used in Oman. In the current study, the INE survey was found to have an acceptable level of internal consistency. Until today there is scanty or no literature about NS incivility in Oman or the Middle East region that can be used to compare with the current findings. Despite its limitations, the study provides important insights into the common forms NS academic incivility and the differences in perception of some of these behaviors between NS and NF.

## Conclusion

During teaching and learning in nursing education both the NF and NS experience real challenging student uncivil behaviors and these can affect the culture of safety and the teaching learning process. The current study found moderate levels of NS academic incivility. The findings also reveal some differences between NS and NF on what is considered to be disruptive behaviors and the incidence of some uncivil behaviors. The moderate incidence of NS academic incivility and the difference in perceptions signals a need for well streamlined policies and strategies to curtail this trend. Academic incivility in nursing education if left untamed can negatively impact knowledge and competence acquisition during training, patient safety during clinical practice, and opportunities to mentor and model NS into responsible professionals.

## Abbreviations

BSN: Bachelor of Science in Nursing
INE: Incivility in nursing education
MSN: Master of Science in Nursing
NF: Nursing faculty
NS: Nursing students
PhD: Doctor of philosophy in nursing
RA: Research assistant
SAQ: Self administered questionnaire
USA: United States of America
